# Evaluation of Silica Nanofluids in Static and Dynamic Conditions by an Optical Fiber Sensor

**DOI:** 10.3390/s20030707

**Published:** 2020-01-28

**Authors:** Marco César Prado Soares, Matheus Santos Rodrigues, Egont Alexandre Schenkel, Gabriel Perli, Willian Hideak Arita Silva, Matheus Kauê Gomes, Eric Fujiwara, Carlos Kenichi Suzuki

**Affiliations:** Laboratory of Photonic Materials and Devices, School of Mechanical Engineering, University of Campinas, São Paulo 13083-860, Brazil; santos.r.math@gmail.com (M.S.R.); egont.schenkel@gmail.com (E.A.S.); perligabriel@gmail.com (G.P.); whideak@gmail.com (W.H.A.S.); matheuskauegomes@gmail.com (M.K.G.); fujiwara@fem.unicamp.br (E.F.); suzuki@fem.unicamp.br (C.K.S.)

**Keywords:** optical fiber sensor, silica nanofluid, concentration measurement, flow speed measurement

## Abstract

This work presents an optical fiber dynamic light scattering sensor capable of simultaneously assessing concentration and flow speed of nanofluids. Silica nanoparticles (189 nm) in water were tested, yielding a sensitivity of 0.78288 × 10³ s^−1^ for static conditions. Then, the sensor was submitted to situations that simulate spatial concentration changes, offering better results than those obtained by traditional mathematical models. Finally, in flow tests, the light backscattered by the nanoparticles were collected by a fiber probe placed parallel to the streamline, whereas intensity values were processed by artificial neural networks. The sensor provides average errors of 0.09 wt% and 0.26 cm/s for concentration and speed measurements, respectively, and can be further applied to assess different types of nanofluids and inline processes.

## 1. Introduction

Nanofluids are stable suspensions of nanoparticles in a base-fluid, and their physical and chemical properties can be tailored according to the characteristics of both dispersed and continuous phases [1]. In particular, silica particles present advantages in terms of sphericity and surface properties; therefore, silica-based nanofluids have been developed for use in thermal conductivity enhancement [2], in-vivo fluorescence imaging [3], and tunable optical filters [4]. In this sense, the assessment of micro and nanoparticles subjected to flow and other dynamic conditions (e.g., concentration gradients and disturbances) is essential not only for silica nanofluids applications, but also for several other biochemical, biomedical, and food engineering uses, such as the monitoring of red cells in blood streams [5], the flow of polymeric particles (e.g., polystyrene) obtained by chemical emulsion processes [6], and the nanoencapsulation of nutrients based on food emulsions [7].

Currently, different techniques are available to measure the properties of nanoparticle suspensions, such as laser Doppler velocimetry (LDV) [6] and particle dynamic analysis (PDA) [8]. However, these approaches rely on bulky and expensive instrumentation, and detection capabilities are limited to concentrations ranging from 10^3^ to 10^4^ particles/mL [9]. There also are electromechanical approaches for the evaluation of the flow of particulate systems, like the sensors for ultrasonic flow assessment and the Coriolis sensor. The ultrasonic sensors are generally based on the use of piezoelectric materials, and rely on transducers, which transform an electric signal into an acoustic wave and vice versa. The acoustic wave must be transmitted across the flow, and will carry information about the whole system. Therefore, it is not invasive, but may be impaired by geometric flaws in the tube and provides only average information (in general, the flow velocity) [10].

In Coriolis sensors, part of the fluid is flown through a U-tube, generating Coriolis forces, which excite an angular motion that is electromagnetically evaluated (i.e., it does not present immunity to electromagnetic interference). These devices are quite expensive and large, thus making it difficult to evaluate mass flows below ~0.5 g/s [11]. Despite the fact that they can be used for evaluation of the density of the flow, proportional to the total of dispersed particles, they are unable to perform colloidal stability analyses, possible with light scattering sensors (when the hydrodynamic diameters of the aggregates are too high due to reduction of stability, they do not scatter light, and no optical signal is detected) [12].

In this context, optical fiber sensors (OFS) are promising alternatives for the aforementioned technologies owing to their intrinsic characteristics, such as compact size, remote operation, and, especially in the case of silica waveguides, immunity to a variety of biochemical reagents [13,14]. For instance, the design of OFS based on the dynamic light scattering (DLS) phenomenon to investigate the size and concentration of static nanoparticles dispersions has been reported in the literature [12,15,16,17].

On the other hand, few studies have addressed the measurement of suspensions under a flow regime by using all-optical fiber schemes. Leung et al. (2006) designed a DLS system in which the fiber probe is inserted perpendicular to the streamline, so the information regarding the particle diffusion coefficient is retrieved from the scattered light intensity and, consequently, correlated to the velocity [9]. Even though the sensor was evaluated for different particle sizes, the system is not capable to resolve the light scattering due to overlaid effects of concentration and velocity. Nilsson et al. (1980), in turn, explored a setup in which the optical fibers are used to launch and collect the light scattered by moving red cells; therefore, it is possible to compare the reference (static) and backscattered signals to detect the Doppler frequency shift, which is related to the flow velocity [5]. However, this technique demands a complex interrogation system to properly retrieve Doppler shift information.

In this sense, the present research proposes an optical fiber DLS sensor for the evaluation of silica nanofluids’ static and dynamic conditions. Firstly, the sedimentation of colloidal silica in test tubes was done to create environments with different concentration zones. The sensor response was evaluated in this case by varying the position of the optical fiber probe along the test tube. Once each zone shows an approximately homogeneous concentration [18], this allows the evaluation of the dynamic response of the sensor in relation to concentration disturbances. The actual concentrations in each zone were calculated by comparing signals with a calibration curve obtained by assessing static suspensions with known silica concentrations. This tests also allowed the obtention of sensitivity.

Finally, the OFS was applied to the detection of velocity of nanofluids with different concentrations and flow conditions. Contrary to previous works, the present system is capable of simultaneously estimating the sample concentration and flow velocity by artificial neural networks, providing a simple, minimally invasive, and nondestructive approach to assess particle dispersions.

## 2. Materials and Methods

### 2.1. Fundamentals

The DLS phenomenon takes place when light interacts with particles whose dimensions are comparable to the radiation wavelength, resulting in absorption and secondary emission in a different direction. In the case of suspensions of nanoparticles in a base fluid, the Brownian motion enhances the dispersion of the collected data; therefore, it is possible to obtain information about the sample by analyzing the characteristics of the scattered waves [19].

Moreover, if the nanoparticles are subjected to flow conditions, there is competition between the fluid translational movement, which exerts drag forces on the particles (viscous action of the fluid that tends to align the particles with the direction of the flow), and the Brownian diffusivity (random motion in all directions) [20].

For independent and non-interacting particles, the homodyne backscattered light intensity I(t) measured by the photodetector in an instant t is function of the total concentration of particles dispersed in the base fluid and of the particles’ diffusion coefficient, D_AB_. If there is a relative translational movement between the nanofluid and the light source, the Doppler shift may also introduce the dependence of I(t) with the flow velocity u and with the beam radius of the source w, where the relation w/u is the beam transit time [9,21].

One may evaluate the autocorrelation function G_2_(τ) of I(t) by Equation (1) [22,23]:

(1) $$G_{2}\left(\tau \right)=\underset{T\to \infty }{\lim}\frac{1}{T}\overset{T}{\underset{0}{\int }}I\left(t\right)\cdot I\left(t+\tau \right)\mathrm{dt}$$

where τ is an arbitrary delay time. G_2_ is a statistical measurement that takes into account the previous history of the system and is influenced by the total number of particles [22,23,24]. Since the light scattering induced by the Brownian motion is influenced by previous particles positions for a sufficiently long time, the phenomenon becomes completely independent and neglects its past history, so G_2_ decreases as τ rises [22].

G_2_ can be approximated by the exponential Siegert relation, Equation (2), where the approximation presents better results for spherical particles [19,25]:

(2) $$G_{2}\left(\tau \right)=\alpha +\beta \cdot \exp\left(-2\Gamma_{m}\tau \right)$$

where α and β are instrumental factors, and Γ_m_ is the average decay rate [19],

(3) $$\Gamma_{m}={\;D}_{\mathrm{AB}}q^{2}$$

where *q* is the magnitude of the scattering vector and D_AB_ is the diffusion coefficient of a particle A in a solvent B. For spherical particles dispersed in low concentrations, D_AB_ can be described by the Stokes-Einstein model [19,20]:

(4) $$D_{\mathrm{AB}}=\frac{\mathrm{kT}}{3{\pi a\mu }_{B}}$$

in which k = 1.38 × 10^−23^ m²⋅kg⋅s^−2^⋅K^−1^ is the Boltzmann constant, T is the system temperature, a is the average diameter of the particles, and μ_B_ is the dynamic viscosity of the fluid. In the presence of a translational movement, Equation (2) must be corrected for Equation (5) [9]:

(5) $$G_{2}\left(\tau \right)=\alpha +\beta \cdot \exp\left(-2\Gamma_{m}\tau \right)\cdot \exp\left(-\frac{u^{2}\tau^{2}}{w^{2}}\right)$$

Finally, the decay rate is determined from the field autocorrelation function G_1_ = G_2_ − α, therefore [19]:

(6) $$\ln G_{1}\left(\tau \right)=\ln\sqrt{\beta }-\Gamma_{m}\tau +K_{2}\frac{\tau^{2}}{2}+K_{3}\frac{\tau^{3}}{6}+\cdots$$

where K_2_, K_3_, … are the moments of distribution of decay rates. Consequently, information about the particles can be retrieved by determining the coefficients of the polynomial (6).

Furthermore, the combination of both diffusion and translation effects can be analyzed in terms of the dimensionless Peclet number Pe [20]:

(7) $$\mathrm{Pe}=\frac{\mathrm{uL}}{D_{\mathrm{AB}}}$$

where L is the tube characteristic dimension. Pe is a relation between the translation and the diffusivity, so it accounts for both effects simultaneously: high Pe values imply in the predomination of the translation over the diffusivity, i.e., the modulation of the Brownian motion by the flow [20].

The symbols mentioned in this research are summarized in Table 1 in the order they are introduced in the text.

It is interesting to analyze the physical interpretation of the abovementioned equations. It is possible to demonstrate that the light scattering vector (Equation (3)) is directly proportional to the total number of particles dispersing the light, and the relation between the decay rate and the particles concentration C is linear for small concentration ranges [19,23,24]. Several works have also empirically observed this linearity [9,12], so a calibration curve correlating Γ_m_ to C can be obtained for a given nanofluid.

The diffusion coefficient may be interpreted as the frequency of the random Brownian motion [19,20]. Therefore, for a given particle diameter (a given diffusivity, according to Equation (4)), an increase of the decay rate is expected as the diameter gets lower. On the other hand, Equation (7) shows that the presence of flow velocity increases Pe and modulates the apparent diffusivity due to predomination of the fluid inertial forces over the random motion, what gives orientation to the particles. Then, as the velocity increases, an apparent reduction on Equation (3) is observed [20].

### 2.2. Sample Preparation

Silica nanoparticles were synthesized by the Vapor-phase Axial Deposition (VAD) method [26], in which a high-temperature O_2_-H_2_ flame promotes the hydrolysis and oxidation of the SiCl_4_ precursor according to the reaction:

(8) $${\mathrm{SiCl}}_{4}{}_{\left(g\right)}+2H_{2}{}_{\left(g\right)}+O_{2}{}_{\left(g\right)}\to {\mathrm{SiO}}_{2}{}_{\left(s\right)}+4{\mathrm{HCl}}_{\left(g\right)}$$

The particles are deposited on the surface of a rotating target, presenting high purity, spherical shape, and amorphous structure. Then, the material is mechanically detached from the target and ground in a quartz mortar to obtain silica powder [12].

The VAD system is designed for the fabrication of completely amorphous silica glass of high transparency, destined for the production of optical fiber preforms and other photonic components [26,27]. Therefore, due to the high purity and morphological control of the silica, and to the wide availability of characterization data of these particles [12,27,28], the use of the in-situ synthesized nanoparticles was preferred over commercially available materials.

Moreover, the morphological control reduces the width of the size distribution and, consequently the effect of the introduction of a particle diameter distribution when performing sedimentation tests. It is also known that a nanofluid system obtained with these particles presents the advantage of not requiring the use of tensoactives to be kept stable, and the particles present high chemical and thermal inertia [12]. An important disadvantage that must be cited, on the other hand, is that the high stability of the nanostructured SiO_2_ dispersion results in a very slow sedimentation when compared to common systems like CaCO_3_, which require only a few hours to be completely sedimented [18].

The nanoparticles were analyzed with a scanning electron microscope (SEM, EVO MA 15, Zeiss, Oberkochen, Germany) equipped with LaB_6_ thermoionic cannon for obtaining the secondary electron images. The samples were prepared by dispersing the SiO_2_ powder in deionized (DI) water, dropping it on a stub, and drying the material under environmental conditions. The sample was gold-coated using a Bal-Tec SCD 050 Sputter Coater to obtain images under high-vacuum conditions. The particles size distribution was measured with a Malvern Zetasizer Nano Zn-Zen 3600 (Malvern Panalytical, Malvern, UK) through LDV.

**Figure 1:**
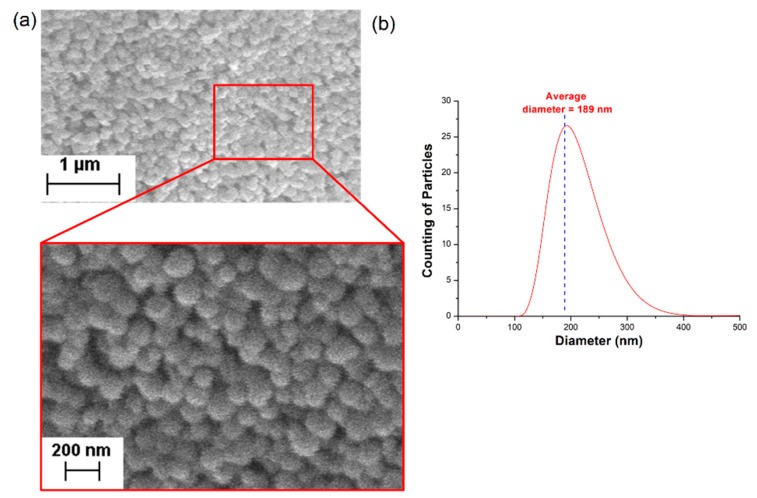
(**a**) SEM image and; (**b**) size distribution of the silica nanoparticles.

The SEM image and the obtained size distribution are shown in Figure 1, yielding an average diameter of a = (189 ± 67) nm, where the half-length of the 95% confidence interval (CI) was obtained by calculating the cumulative integral of the size distribution and then taken as the uncertainty of the diameter. It is worth noticing that, once the particle distribution evaluation provides a hydrodynamic diameter, which includes at least one layer of solvent molecules around the nanoparticles, this average diameter may be slightly larger than that observed in the SEM image.

To obtain the SiO_2_ suspensions, nanoparticles were dispersed in DI water according to a mechanically mixing procedure, followed by sonication in a temperature-controlled ultrasound bath (5.9 L Ultrasound Bath, Ultronique Eco-Sonics, Indaiatuba, SP, Brazil) for 180 min to ensure homogenization. The samples present a practically constant dynamic viscosity value of μ_B_ = 0.933 × 10^−3^ kg⋅m^−1^⋅s^−1^ at 25 °C [12], so the diffusivity calculated by Equation (4) is given by D_AB_ = 2.48 × 10^−12^ m^2^.s^−1^ at 25 °C.

### 2.3. Optical Fiber Sensor

**Figure 2:**
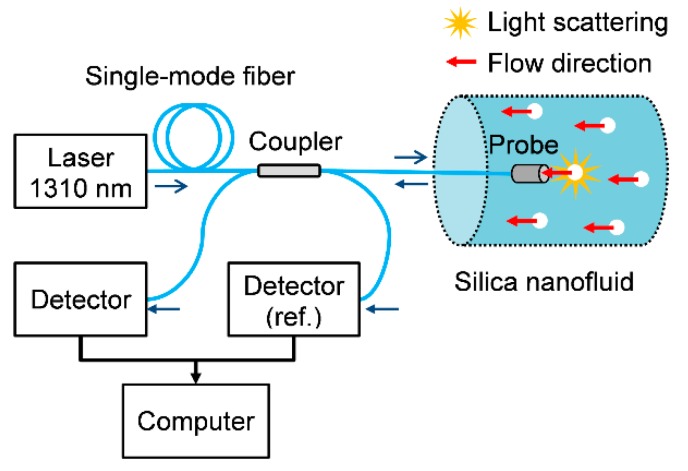
Optical fiber DLS sensor setup.

The OFS is depicted in Figure 2 [29]. Light emitted by a 1310 nm laser diode is launched into the standard silica single-mode fiber (SMF). The optical signal is divided by a 1 × 2 90:10 directional coupler: 10% of the light is used as the reference whereas the remaining part is delivered to the sample. The fiber end-face (plane-polished and encapsulated in zirconia ferrule) works as the probe. Therefore, reflection occurs at the fiber-liquid interface due to the refractive indices difference according to the Fresnel equation [22]:

(9) $$I\left(t\right)={\mathrm{cI}}_{R}{\left(\frac{n_{c}-n_{s}}{n_{c}+n_{s}}\right)}^{2}$$

where I(t) and I_R_ are the reflected and the reference light intensities, respectively, c is a coupling coefficient that accounts for power losses, and n_c_ and n_s_ are the refractive indices of the fiber core and the sample, respectively. Finally, the reflected light is guided to the photodetector and processed in a computer.

For the nanoparticle suspension, the backscattered light is also partially coupled to the fiber core, so the DLS effect emerges as a superimposed noise in the I(t) signals. Consequently, one may evaluate ln(G_1_) to retrieve Γ_m_, and then investigate particle concentration and flow velocity. The optical signal is acquired at 1 kHz sampling rate and processed by MATLAB (Mathworks) routines [29].

To create an environment with different particles concentrations (suitable for the simulation of concentration disturbances), two (1000 mL)-test tubes were filled with silica suspensions of 1 wt% of concentration. The suspensions were left in rest so the particles could slowly sediment, creating a clarified zone on the top of the flask, a concentrated zone on the bottom, and zones with intermediate concentrations between these two [18]. During all of the sedimentation time, the tubes were kept sealed to avoid contamination with external microorganisms, and microscope images were obtained for all of the zones at the end of the experiment.

For the sensor experiments, the optical fiber probe was immersed in one of the regions to collect 20,000 optical data, and then rapidly moved to another zone, where its response was evaluated again. All experiments were performed at room temperature (~25°C).

The height h(t) from the bottom of the tube to the beginning of the clarified zone presents an initial value h_i_ that progressively decreases with time. If the initial concentration of particles is C_i_ (mass/volume), and assuming that the concentration in the clarified zone is negligible (so that the particles are all on the most concentrated zone), the estimated concentration C(t) (on the bottom zone) is given by Equation (10), a direct consequence of the mass balance of solid particles [18].

(10) $$C\left(t\right)=C_{i}\frac{h_{i}}{h\left(t\right)}$$

**Figure 3:**
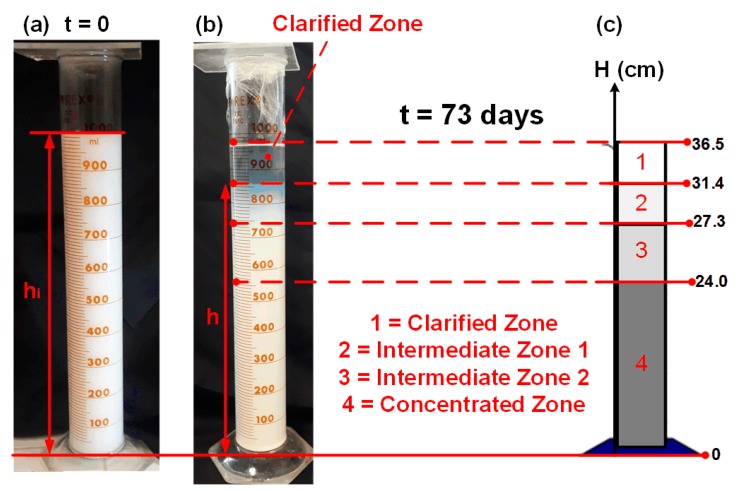
Test tube containing the colloidal silica: (**a**) first day of experiment; (**b**) 73 days after the beginning of sedimentation; (**c**) 4 different sedimentation zones that could be distinguished on day 73. Each zone presents a different value of particle concentration, creating an environment where it is possible to simulate concentration disturbances (heights expressed in cm units).

Figure 3 shows one of the test tubes on the first day of experiment and after 73 days of sedimentation, when 4 concentration zones are distinguishable. This long time required for the visualization of these zones is a consequence of the abovementioned low average diameter and size distribution width and very high suspension stability of the SiO_2_ nanofluid, resulting in a slow introduction of size distribution.

It is important to also notice that the half-width of the 95% CI of the sizing distribution shown in Figure 1b is of only 67 nm. It is known that, for SiO_2_ nanoparticles with diameters of this order of magnitude (from ~125 to about 200 nm), this diameter difference is only expected to result in a significant difference in decay rate signals of light scattering sensors like the one used in this research for concentrations higher than 1.5 wt% (almost no difference is expected for values lower than 1.2 wt%) [12]. This is a consequence of the fact that the autocorrelation function (Equation (1)) is a statistic that takes into account all of the intensity measurements performed, and whose quality depends on the total number of particles scattering the light. In addition, the small number of particles present for concentrations lower than 1.2 wt% reduces sensitivity [22], and no differences regarding the average diameter can be noticed [12]. Therefore, sedimentation tests must be performed for concentrations in this range, such that the interference of this factor will not affect the analysis. Indeed, the application of the heights shown in Figure 3 on Equation (10) results in an estimated concentration of only ~1.16 wt%; thus, the tube was considered adequate for this research.

The probe was sequentially dislocated from the clarified (Zone 1) to the more concentrated zone (Zone 4), and then the opposite sequence was performed. Each zone change is equivalent to a step-disturbance on nanoparticle concentration.

To correlate the DLS with actual concentration of particles in each zone (that may not be equal to the model predictions), a calibration curve was obtained. The optical fiber is introduced into nanofluids with different concentrations ranging from 0 (DI water) to 2.0 wt%, and the sensor static response and its sensitivity in relation to the concentration are evaluated.

Finally, the nanofluids were evaluated when submitted to the flow regime. In this case, the probe is placed parallel to the nanofluid flow streamline (as shown in Figure 2), so the light scattered by particles is coupled to the fiber and measured with a photodetector. The nanofluids with different concentrations C are moved through a 6-mm inner-diameter silicone tube under hydrostatic pressure gradient at room temperature, while the flow speed u is controlled by a microvalve and the optical response is analyzed using the fiber sensor.

The experiments were carried out for SiO_2_ suspensions with concentrations C ranging from 0.50 wt% to 2.00 wt% and velocities u ranging from 0 to 9.5 cm/s. Taking the characteristic dimension L as the tube diameter and D_AB_ as the value calculated in Section 2.2, Equation (7) shows that Pe is directly proportional to u, and is simply given by Pe = (2.42 × 10^7^) u, u in cm/s. Therefore, u and Pe can be easily exchanged by simple conversion, and all of the conclusions regarding the increase of u can be extended for the increase of Pe (i.e., the increase of the flow inertial forces in relation to the diffusion of the nanoparticles).

It is also important to notice that, for obtention of the calibration curve and for the experiments that simulate concentration disturbances in the test tubes (in which the probe is dislocated to each zone and is maintained in the same position until it completes 20,000 measurements), the velocity of the fluid u is zero and the second exponential term in Equation (5) is equals to 1. For the experiments with nanofluids flowing, on the other hand, the second exponential increases with velocity u [9], introducing a non-linearity into the relation between Γ_m_, u, and C.

## 3. Results and Discussion

**Figure 4:**
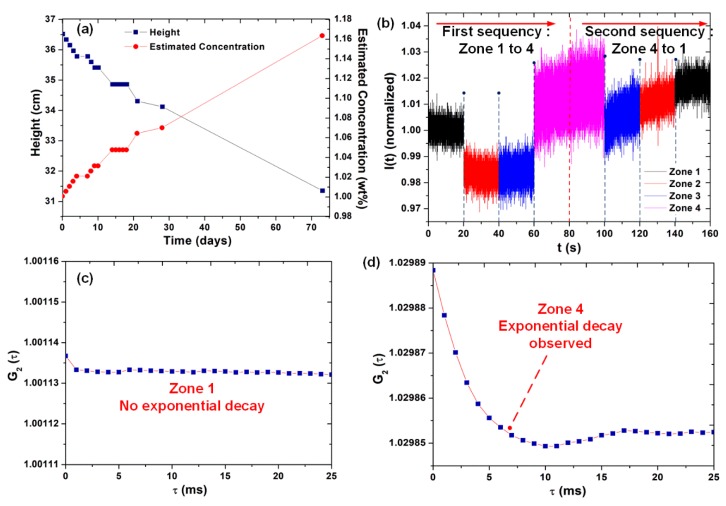
(**a**) Average values of the heights verified for the two tubes and concentrations estimated from Equation (10) (secondary vertical axis); (**b**) sequential step disturbances in the concentration of the OFS probe; (**c**) G2(τ) obtained for Zone 1, as function of the arbitrary delays applied by the algorithm; (**d**) G2(τ) obtained for Zone 4, with an exponential decay, as function of the arbitrary delays applied by the algorithm.

Figure 4a shows the heights h of the end of the clarified zone over the 73 days of the experiment and the respective concentrations estimated by Equation (10). The OFS was used to evaluate the clarified zone during the experiment, but no DLS phenomenon was observed, suggesting that the zone is approximately free of particles.

The sensor probe was sequentially moved from Zone 1 to Zone 4 (from concentration of 0 to 1.16%, according to Equation (10)), in a first sequence of step disturbances, and then moved in a second sequence, from Zone 4 to Zone 1 (Figure 4b). Figure 4c shows G_2_(τ) obtained for Zone 1 (clarified), where no exponential decay is observed (no DLS detected). Figure 4d, in turn, shows G_2_(τ) obtained for Zone 4, with the exponential decay characteristic from the scattering (Equations (2) and (5)): when there are dispersed particles, the autocorrelation function shows an exponential decay followed by the achievement of a baseline, and the rate of this decay is correlated to their concentration. In many experiments, however, a minimum point of the function or an oscillation of the baseline may be observed, and they are caused by experimental deviations or by the discretization errors involved in the mathematical processing of the signal by the algorithm in Equations (1)–(6) [12,19]. This minimum point and baseline oscillation are observed in Figure 4d and, due to their presence, a satisfactory polynomial regression (Equation (6)) is required for the correct evaluation of Γ_m_.

**Figure 5:**
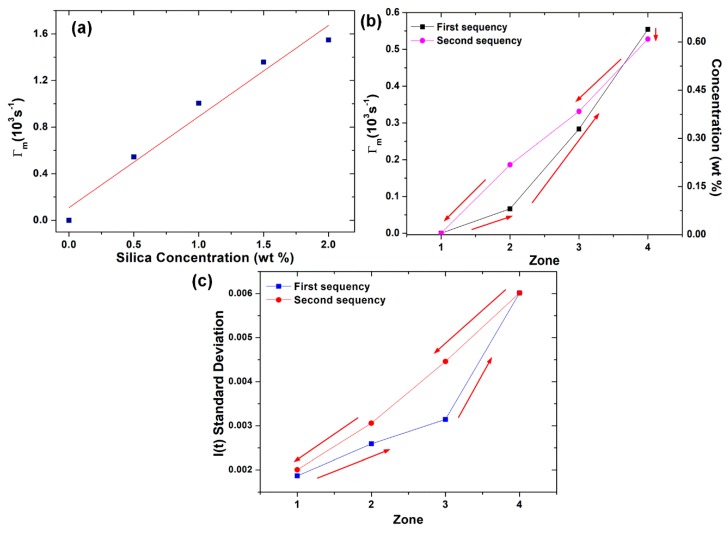
(**a**) Calibration curve; (**b**) Γ_m_ and concentrations; (**c**) standard deviations in each zone.

In order to assess the concentration in each zone, a calibration curve was obtained by calculating Γ_m_ for suspensions with known concentrations of silica, Figure 5a. The linear regression of the experimental data resulted in Γ_m_ = 0.78288C + 0.10898, C in wt% (R² = 0.95942), yielding 0.78288 × 10³ s^−1^ sensitivity. Figure 5b shows the Γ_m_ calculated for each zone and the concentration calculated with the calibration curve, and Figure 5c shows the standard deviations σ of the signals collected for each zone.

It is possible to notice that the actual concentration on Zone 4 is quite inferior than the one predicted by Equation (10), and its value is on the range where diameters differences (induced by the sedimentation) are not expected to interfere with the decay rate evaluation (detected concentration of ~0.57 wt%).

McCabe et al. [18] and Foust et al. [30] recognize that the majority of sedimentation vessels result in the formation of sedimentation zones, but, for simplifying the engineering project of the sedimentation equipment, they use a mathematical model that considers the presence of only two zones (Equation (10)), the clarified and the concentrated bottom zone. Since Figure 3 shows that there are four different zones with different concentrations, part of the particles that Equation (10) predicted to be in Zone 4 are divided between Zones 2 and 3, so the actual concentration of Zone 4 is lower than predicted. The sensor solves this problem, since it provides a simple and more accurate evaluation of C.

The results also show the same tendencies between Γ_m_ and σ, with similar behavior for both sequences. Figure 5b shows small modifications of the average signal when the probe is moved from one zone to another, probably caused by fiber macrocurvature losses. It is also possible to evaluate the lowest and highest signal-to-noise ratios (SNRs) as μ²/σ², where μ is the mean value of the collected signals [22]: 2.604 × 10^4^ (Zone 4, second sequence) and 2.869 × 10^5^ (Zone 1, first sequence), respectively. The lowest SNR for Zone 4 is a direct consequence of the increase of nanoparticles concentration, since the DLS phenomenon results in the enhancement of light intensity dispersion [29].

When analyzing Figure 5b,c, a hysteresis can be noticed, but this effect is lower for Figure 5b (the signal differences between measurements taken for same concentration zones are lower when evaluated in terms of decay rate). A probable cause is the difference between the statistical methods applied to each figure. The results obtained with the autocorrelation function take into account not only the whole group of measurements, but also the memory effect of the light intensity oscillations related to the Brownian motion (i.e., the fact that each oscillation of the particle in a given moment affects the immediately following oscillations, but not the ones separated by large temporal distances) [19,22]. On the other hand, it is known that decay rate measurements are not dependent on the particular light intensity reference adopted by the photodetection system [19], which is not true for the standard deviation quantification. Despite these disadvantages, the computational cost and time required for the calculation of σ are lower, which can make the standard deviation evaluation more attractive.

Moreover, the elucidation of the physical cause of the hysteresis requires more investigation. Feasible hypotheses are that: when pulling back the probe to the surface of the flask, the environment is disturbed, carrying particles from the lower to the upper zones; or that a small quantity of particles remains close to the probe surface due to the inertial forces of the optical fiber movement and to the surface attraction between the two silica materials.

**Figure 6:**
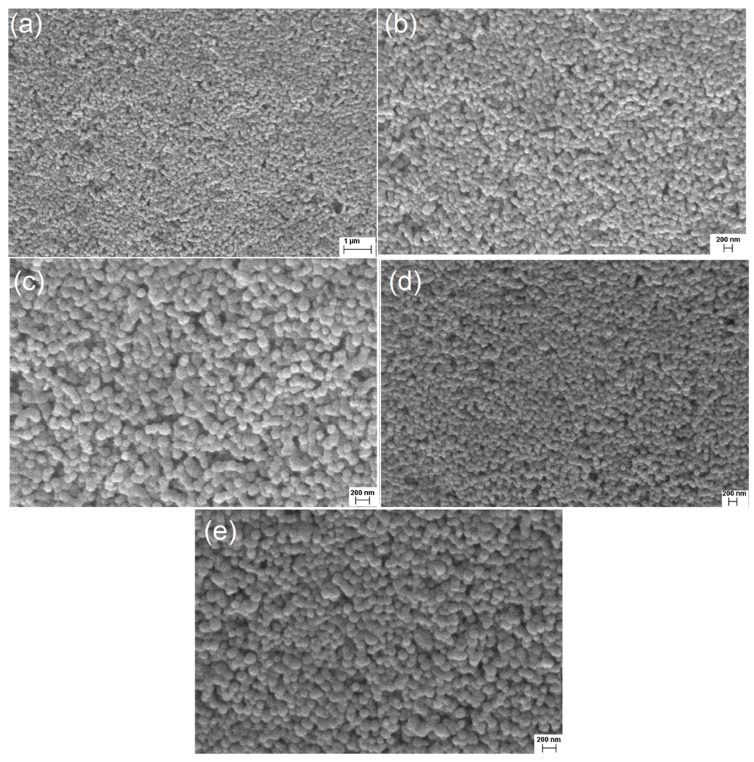
(**a**) General aspect of the most concentrated zone (image useful for the detection of particles with diameters much higher than the SiO_2_); (**b**) detail of particles collected in Zone 1; (**c**) detail of particles collected in Zone 2; (**d**) detail of particles collected in Zone 3; (**e**) detail of particles collected in Zone 4. It is difficult to observe significant differences between the average diameters of the images, and no microorganisms were noticed.

Figure 6a reveals the general aspect of the material deposited on the bottom of the flask, whereas Figure 6b–e show microscopic images obtained for particles collected in each sedimentation zone under higher magnification. It is difficult to visually observe differences in the average diameters, as predicted (the nanofluid stability and the time required to visualize the sedimentation zones are very high, so only a significantly low sizing distribution is introduced). The images also do not show the presence of algae or other microorganisms. These cells are usually observed as particles with diameters in the order of ~1 μm [29].

The presence of these cells, on the other hand, would probably not be a significant problem for the sensor analysis: the decay rates collected with this type of sensor for yeast cells of *Saccharomyces cerevisiae* are usually much lower than the ones in Figure 5a, and may be one order of magnitude lower for concentrations of cells up to 1 × 10^8^ cells/mL (a concentration where the cells are easily detected by microscopes and usually observed on the bottom of the flasks as a yellow or green precipitate) [29]. These low signals collected for them are a direct consequence of Equations (3) and (4): the very higher diameters of these cells in relation to the SiO_2_ particles (1 μm against ~200 nm) result in lower diffusivities (lower Brownian motion frequencies), leading to smaller decay rates.

**Figure 7:**
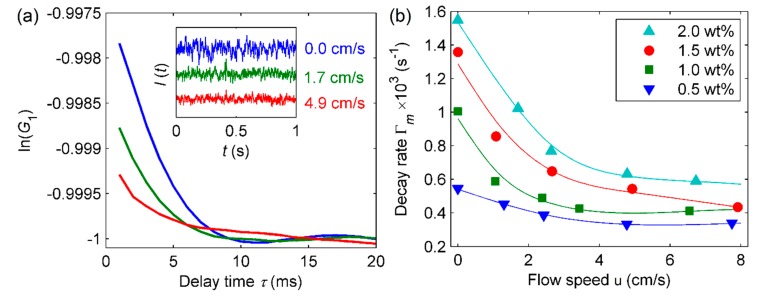
(**a**) Normalized autocorrelation function for 2 wt% silica nanofluids at different flow speeds. The inset shows the normalized scattered light intensities; (**b**) Γ_m_ as a function of flow speed for different concentrations. The solid lines are guides to the eye.

The results collected for the evaluation of flow conditions are shown in Figure 7. The scattering increases for concentrated solutions due to the particle Brownian motion, but decreases with speed since the light intensity is attenuated by longitudinal flow dynamics and laser beam depth resolution [31]. This explains the decay rate Γ_m_ reduction with u, in contrast to that observed in transverse flows [9].

The influence of u in the light scattering can be explained by the competitive effects between the Brownian and translational motions. As the flow velocity increases (increase of Pe), the translational component of the particles position vector overcomes the diffusive effects and modulates the magnitude of I(t) [20,24]. This is observed in Figure 7b: as the inertial forces become dominant with the increase of Pe, an approximate constant decay rate is reached, indicating the decrease of the influence of the Brownian diffusivity. However, the diffusion effect is not completely eliminated, since Figure 7b shows that the final decay rate is higher for more concentrated nanofluids.

When comparing Figure 7b with Figure 5a (static case), it can be noticed that the relation between the light scattering, flow speed, and particle concentration is highly non-linear. It is a consequence of the increase of the second exponential term in Equation (5) with u, as already mentioned [9]. Therefore, in order to achieve a precise mathematical model instead of using successive linear interpolations, data analysis with Artificial Neural Networks (ANN) was proposed. ANN allow the obtention of more precise results without losing the generality of the phenomenon for the tested range, a characteristic that is highly attractive for the development of new instrumentation [32].

Furthermore, to evaluate concentration and flow speed simultaneously, an ANN correlator was implemented in MATLAB by varying the delay step Δτ from 100 to 1000 ms [33] using the k-fold cross-validation method [32,34]. The measurements were randomly selected without reposition to form k = 10 distinct groups with six measurements (k-value justified by the amount of data and by the fact that the measurements were relatively sparse). After this division, the ANN was trained with k_i-1_ groups and tested with the ith-group k_i_, for all of the 10 groups i = 1, 2, …, 10, a process known as “leave-one-out”. Despite the fact that the error estimator’s variance and the computational effort are proportional to the number of folds, the use of 10 folds results in a low-biased and highly-accurate estimator, in contrast with other traditional training methodologies, like the “Bootstrap” method [34].

The size of the ANN, in turn, was defined to avoid overfitting: the number of layers was fixed to 2 (only one hidden layer) and the number of neurons was progressively increased. The increase was performed until the error achieved a value considered satisfactory (when the increase on the number of neurons no longer produced a significant reduction of the error), and without the occurrence of overfitting.

**Figure 8:**
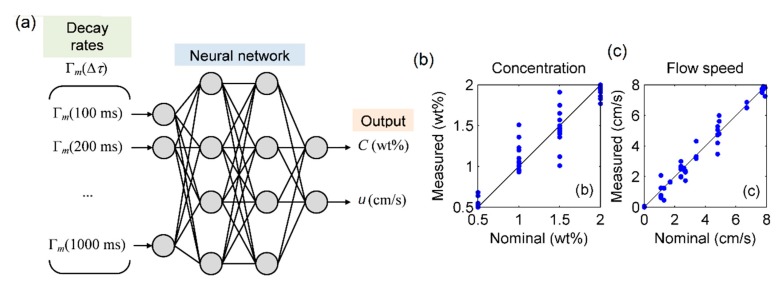
ANN correlator: (**a**) neural network architecture; and measurement of errors for (**b**) concentration; and (**c**) flow speed.

Then, an array of decay rates Γ_m_(Δτ) was used as the input of 2 layers, 20 neurons per layer, using the backpropagation artificial neural network with tangent sigmoidal transfer function, returning C and u. The network was trained with scaled conjugate gradient algorithm using 10-fold cross-validation [33,34]. The general architecture of the ANN and the results obtained for these parameters are shown in Figure 8, yielding 0.09 wt% and 0.26 cm/s average absolute errors for concentration and flow speed, respectively.

**Figure 9:**
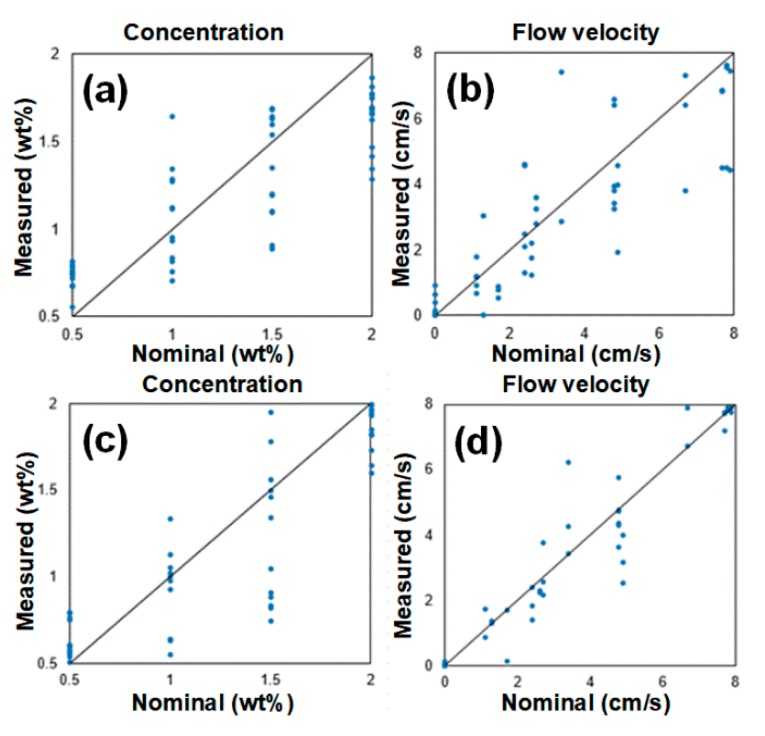
ANN correlator: measurement errors for (**a**) concentration (16 neurons); (**b**) flow speed (16 neurons); (**c**) concentration (18 neurons); and (**d**) flow speed (18 neurons).

Figure 9 shows the results that could be obtained for slightly lower values of neurons. It is easily noticed that the use of less than 20 neurons results in a considerable increase in error: average absolute errors of 0.44 wt% and 1.15 cm/s for concentration and flow speed, respectively (when using 16 neurons); and average absolute errors of 0.37 wt% and 0.88 cm/s for concentration and flow speed, respectively (18 neurons).

The results collected for the correlator using 20 neurons are comparable to that reported for optical coherence tomography [31], laser Doppler velocimetry [19], and conventional DLS [35] systems. The proposed method presents several advantages over these studies, since it provides simultaneous measurements and relies on simple, low-cost, all-optical fiber instrumentation, which is suitable for inline nanofluids monitoring.

The simplicity and low cost of the system, on the other hand, does not allow the evaluation of particle size distribution, since it does not retrieve angular information regarding optical signals.

## 4. Conclusions

An optical fiber sensor was successfully applied to the monitoring of a multi-concentrated particulate system (silica nanofluid) by creating an environment suitable for the simulation of concentration step disturbances. The information collected with the system show that the real concentration values are very different from the ones estimated by the simple sedimentation model traditionally applied, which is of great practical importance. The reason for this difference is an assumption of the model: in order for simplifying engineering projects of sedimentation tanks (applied to important environmental procedures), the model only considers the presence of two sedimentation zones: the clarified and the concentrated bottom zone. This results in empirical differences, since the height of the more concentrated zone is actually lower than the end of the clarified, and the particles are divided between intermediate regions. On the other hand, it is not possible to simply apply the height of the most concentrated zone to Equation (10), since the simple proportion expressed in this equation is derived from the mass balance between only two different regions [18].

Once this model is used for industrial equipment projects and for their monitoring during operation [18], more precise information can save fabrication costs and allow the obtention of more efficient equipment.

Finally, a DLS sensor based on an ANN correlator was demonstrated for the simultaneous assessment of concentration and flow speed in silica nanofluids. Moreover, this optical system is further applicable to other types of nanoparticles and inline processes, especially for applications in industries and laboratories that works with particulate liquid systems - from the control and monitoring to the engineering projects.

## Figures and Tables

**Table 1:** Mathematical symbols given in the order they are introduced in this paper.

Symbol	Meaning	Units
I(t)	Light Intensity Detected in a Given Instant	−
t	Time	s
D_AB_	Diffusion Coefficient	cm²⋅s^−1^ or m²⋅s^−1^
*u*	Flow Velocity	cm⋅s^−1^
w	Light Source Beam Radius	cm
G_2_	Autocorrelation Function of I(t)	−
τ	Arbitrary Delay Time	s
α and β	Fitting Parameters	−
Γ_m_	Decay Rate of the Autocorrelation	s^−1^
**q**	Magnitude of the Light Scattering Vector	cm^−1^
k	Boltzmann Constant	m²⋅kg⋅s^−2^⋅K^−1^
T	Temperature	K
a	Average Diameter of Particles	m
μ_B_	Dynamic Viscosity of the Fluid	kg⋅m^−1^⋅s^−1^
G_1_	Field Autocorrelation Function of I_R_	−
K_2_, K_3_, …	Moments of Distribution of Decay Rates	s^−2^, s^−3^, …
Pe	Peclet Number	−
L	Characteristic Length of the Flow	cm
I_R_	Reference Light Intensity	−
c	Coupling Coefficient	−
n_c_	Refractive Index of the Fiber Core	−
n_s_	Refractive Index of the Sample	−
h(t)	Height from the Bottom to the Clarified Zone	cm
h_i_	Initial Value of h(t)	cm
C_i_	Initial Concentration of Nanoparticles	g⋅L^−1^ or % (mass per mass)
C(t)	Instant Concentration of Nanoparticles	g⋅L^−1^ or % (mass per mass)
σ	Standard Deviation of the Collected Signals	−
μ	Mean Value of the Collected Signals	−
